# The Living-Out System

**Published:** 1920-12-18

**Authors:** 


*To the Editor of* The Hospital.

### The Living-out System.

*To the Editor of* The Hospital.

Sir,—I have read with much interest the article in the Matrons' and Sisters' column on the system of living out. Something certainly will have to be done, and there is little doubt but that this "something" hinges on the question of "£ s. d." Take, for instance, a ward sister who has been nursing for ten years: her salary is possibly £60 per annum, with board, lodging, washing, and uniform. I wonder what monetary value would be given to her for these emoluments? It is quite certain that whatever value is placed upon these emoluments by the hospital authorities, the equivalent in value could not be purchased by her for that sum. Do the majority of nurses truly desire to live out? I think not. Unless salaries are greatly enhanced, it would be impossible for nurses to live out, except in rare instances where it happens that the nurse's parents live in the vicinity of the hospital. To live out must not mean the unutterable misery of a bed-sitting-room in somebody else's house; the freedom of a nurses' home in a hospital would, I think, be infinitely more desired by the majority of nurses whom I have been in contact with. My experience during the war, when for about two years some sixty members of our staff were billeted out, was that the nurses as a whole preferred to live in. They missed the excellent supply of hot water, the possibility of having a bath at any hour, night or day. It is true that they had their own latch-key, but it is much easier and more comfortable to return to a hospital after a theatre pass than to return to ultra respectable suburbia. To quote an instance: During the war a night nurse received permission from the night sister to go off duty at 6.30 a.m., as she had not been well. This was construed by the landlady into "the nurse had stayed out all night." The restrictions of having a room in somebody else's house are far more irksome than living in a nurses' home in hospital, and the life infinitely more lonely. To be able to take a broad view of life and to keep in touch with a wide circle, and to realise the interdependence of each stratum of life, requires the assurance of sufficient income, without undue anxiety for the future. Certainly much could be done to improve the living-in system. It could be a hostel, where each fully-trained nurse had her own bedroom and sitting-room, built in the form of a flat; bathrooms and lavatories common to all. The rooms should be heated and supplied with a gas-ring and slot meter; the dining-room somewhat on the restaurant system, with a trained housekeeper solely responsible for the catering in all its branches; a room fitted up and set apart where the staff who felt so disposed could do some washing and ironing for themselves. Building, I know, is costly, but the continual "tinkering at" and "making do" with existing conditions, which it has been my experience to witness, is, I am sure, far costlier still, and leaves us in exactly the same unsatisfactory position.

It would be interesting to know what salaries are considered adequate by the advocates of the living-out system.—Yours faithfully,

December 8, 1920. Cornubia.

